# Ag_2_S/Zn^2+^-Decorated g-C_3_N_4_ Type-II Heterojunction with Wide-Spectrum Response: Construction and Photocatalytic Performance in Ciprofloxacin Degradation

**DOI:** 10.3390/molecules30071417

**Published:** 2025-03-22

**Authors:** Chengyang Wang, Han Zheng, Ruxue Ma, Xiucheng Zheng, Xinxin Guan

**Affiliations:** 1 College of Chemistry, Zhengzhou University, Zhengzhou 450001, China; 2 State Key Laboratory of Coking Coal Resources Green Exploitation, Zhengzhou University, Zhengzhou 450001, China

**Keywords:** Zn^2+^-decorated graphitic carbon nitride, silver sulfide nanoparticles, broad-spectrum responsive type-II heterojunction, ciprofloxacin degradation, universality

## Abstract

Antibiotic-based wastewaters seriously endanger human health and damage the ecological environment, and photocatalytic degradation is a desirable strategy for eliminating these contaminants in water. Therefore, developing a proper catalyst for the photodegradation of antibiotics, including ciprofloxacin (CIP), is of great importance. In this study, novel Ag_2_S/Zn^2+^-decorated graphitic carbon nitride (AZCN for short) type-II heterojunctions are constructed through a precipitation–calcination procedure. The high porosity with a specific surface area of 133.5 m^2^ g^−1^, as well as the positive synergy between Ag_2_S- and Zn^2+^-decorated graphitic carbon nitride (abbreviated as ZCN), enhance incident light harvesting, increase the adsorption capacity for reactant molecules, favor mass transfer and promote the separation and transport of photoinduced carriers, therefore improving the degradation efficiency of CIP. Specifically, the degradation efficiency of CIP (50 mL, 10 mg L^−1^) over 2.5% AZCN (10 mg) is 18.1%, 43.1% and 55.7% within 60 min of irradiation using near-infrared light, visible light and simulated solar light, respectively. Moreover, it displays satisfactory recycling stability and excellent universality. This research not only develops a promising heterojunction photocatalyst but also offers some valuable insights in water remediation.

## 1. Introduction

In line with the rapid development of society, antibiotics have been used in diverse domains, such as anti-inflammatory and sterilization interventions, as well as livestock and poultry farming [1,2]. However, the widespread adoption of antibiotics has led to huge residues in the environment. These harmful residues come from pharmaceutical factories, hospitals and domestic use, as well as livestock and poultry farming effluents, which can pollute surface water and groundwater, endangering human well-being and ecological systems [3,4]. Specially, as a significant second-generation fluoroquinolone antibiotic, ciprofloxacin (CIP) is a very effective antimicrobial and anti-inflammatory drug and has been widely adopted. As a result, its concentrations in wastewater have reached even up to 50 mg L^−1^ [5]. In addition, conventional wastewater treatment plants cannot completely eliminate antibiotics. Hence, it is worthwhile to develop an effective and environmentally friendly antibiotic elimination technology.

In recent decades, photocatalytic degradation has been verified to be a promising technology for eliminating antibiotics in water by efficiently using sustainable solar light. Notably, this strategy has exciting advantages, such as eco-friendly operation, broad applicability for diverse organic contaminants, selective targeting and complete mineralization of the substrate molecules [6]. Considering the imperative role of catalysts in photocatalysis, many scholars have focused on photocatalysts for antibiotic degradation, such as those summarized in recent reviews [7,8,9,10,11,12,13,14]. Even so, developing a novel photocatalyst is still challenging and attractive because of the unsatisfactory practicality of the reported materials.

Unlike metal-based materials, the non-metallic n-type polymer semiconductor graphitic carbon nitride (g-C_3_N_4_) can avoid metal residue-related secondary pollution when used as a photocatalyst. Moreover, apart from having decent thermal and chemical stability, unique optical properties and exciting biocompatibility, g-C_3_N_4_ is non-toxic, harmless and can be easily synthesized from various precursors. Therefore, it has become a popular research focus in photocatalysis [15,16,17,18]. However, the poor electron conductivity, low utilization of visible light and quick recombination of photoinduced carriers hamper the practicality of bulk g-C_3_N_4_. To impressively resolve these obstacles and improve photodegradation efficiency, the researchers have tried various modification strategies for g-C_3_N_4_, such as introducing N defects or proper dopants, as well as constructing suitable heterojunctions or compounding with other semiconductor materials. For example, Liu et al. prepared N-defective g-C_3_N_4_ by using NaBH_4_, which can offer an approximately ten-fold increase in the rate constant of CIP degradation in comparison with pristine g-C_3_N_4_ [19]. Ma et al. prepared S-type 2D/2D g-C_3_N_4_/NH_4_V_4_O_10_ heterojunctions by intercalating g-C_3_N_4_ into ultrathin NH_4_V_4_O_10_ nanosheets, and the elimination rate of CIP (10 mg⋅L^−1^) over 50-CNNS/NH_4_V_4_O_10_ was 92% under simulated sunlight illumination [20]. Chankhanittha et al. prepared the Z-scheme g-C_3_N_4_/BiOBr/Bi_2_MoO_6_ heterojunction using a one-pot hydrothermal method, which offered a CIP degradation efficiency of 94% within 180 min of irradiation using visible light supplied by a LED lamp [21]. Sohaimi et al. found that the CIP removal efficiency was as high as 91% during the daytime and 75% at night in the presence of a V_2_O_5/_g-C_3_N_4_ composite with a 2% loading ratio [22]. Niu et al. reported that the CIP degradation efficiency was improved 26.1% when loading a BiVO_4_/g-C_3_N_4_ photocatalyst on carbon paper (CP) due to reduced electron-transfer resistance [23]. Nonetheless, few articles have been published about g-C_3_N_4_-based photocatalysts prepared by simultaneously using several modification strategies for CIP degradation.

In our previous work [24,25], we developed porous N-defective g-C_3_N_4_ (abbreviated to CN in this study) via the co-polymerization of melamine and urea and then optimized the Zn^2+^-doped CN photocatalyst (abbreviated to ZCN in this study) using an ultrasound treatment–calcination method. Both materials displayed exciting photocatalytic performances in degrading certain organic contaminants (e.g., rhodamine B dye as well as a tetracycline antibiotic) under visible light irradiation. To increase the usage of solar light and specially develop a novel photocatalyst for eliminating the CIP antibiotic in water, inspired by the aforementioned knowledge, we further couple ZCN with Ag_2_S nanoparticles (NPs) by using the simple co-calcination method for CIP degradation. The resulting optimal type-II heterojunction is characterized in detail, and a range of experiments under different conditions were employed for exploring its structural–photocatalytic performance relationships, photocatalytic mechanism and universality in purifying organic wastewaters. This work is expected to contribute to the development of heterojunctions in photocatalysis.

## 2. Results and Discussion

**Figure 1:**
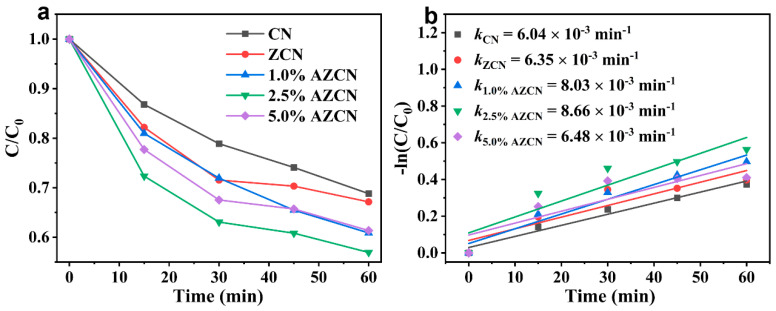
Photocatalytic activities of CN, ZCN and *x*% AZCN (10 mg) in the degradation of CIP (50 mL, 10 mg L^−1^) irradiated using visible light (using 420 nm cutoff filter) (**a**) and the corresponding curves of −ln(C/C_0_) vs. irradiation time (**b**).

The photocatalytic activity of *x*% AZCN was investigated in CIP degradation under visible light illumination and compared with that of CN and ZCN. Our control experiment has verified that CIP self-degradation is ignorable in the absence of any photocatalyst, while all the as-prepared materials exhibit significant photocatalytic activity under current reaction conditions (Figure 1a). Furthermore, *x*% AZCN displays higher photocatalytic activity than CN and ZCN, and the decontamination efficiency of CIP increases with a properly increasing Ag_2_S amount in the resulting heterojunctions. However, the degradation efficiency decreases when further increasing the amount of Ag_2_S. Additionally, 2.5% AZCN poses the highest activity among the investigated materials, and the degradation efficiency of CIP is 43.1% within 60 min. As shown in Figure 1b, based on the well-accepted pseudo-first-order kinetic model, the reaction rate constant (*k*) over this heterojunction is 8.66 × 10^−3^ min^−1^, strikingly higher than that over CN (6.04 × 10^−3^ min^−1^), ZCN (6.35 × 10^−3^ min^−1^), 1.0% AZCN (8.03 × 10^−3^ min^−1^) and 5.0% AZCN (6.48 × 10^−3^ min^−1^). In addition, the adsorption of the CIP experiment under dark conditions showed that the 2.5% AZCN had the strongest adsorption ability of CIP among the as-synthesized catalysts (Figure S1). Namely, 2.5% AZCN is the optimal heterojunction photocatalyst in this study. Although the resulting degradation value (43.1%) is not outstanding in comparison with the recent reported results summarized in Table 1, considering the adopted reaction parameters, especially the photocatalyst dosage, illumination light type and time, 2.5% AZCN still possesses a certain competitiveness in purifying CIP-based wastewater. Therefore, this heterojunction is characterized in detail and applied in the subsequent photocatalytic experiments to study its structure–performance relationships, photocatalytic mechanism and universality.

**Figure 2:**
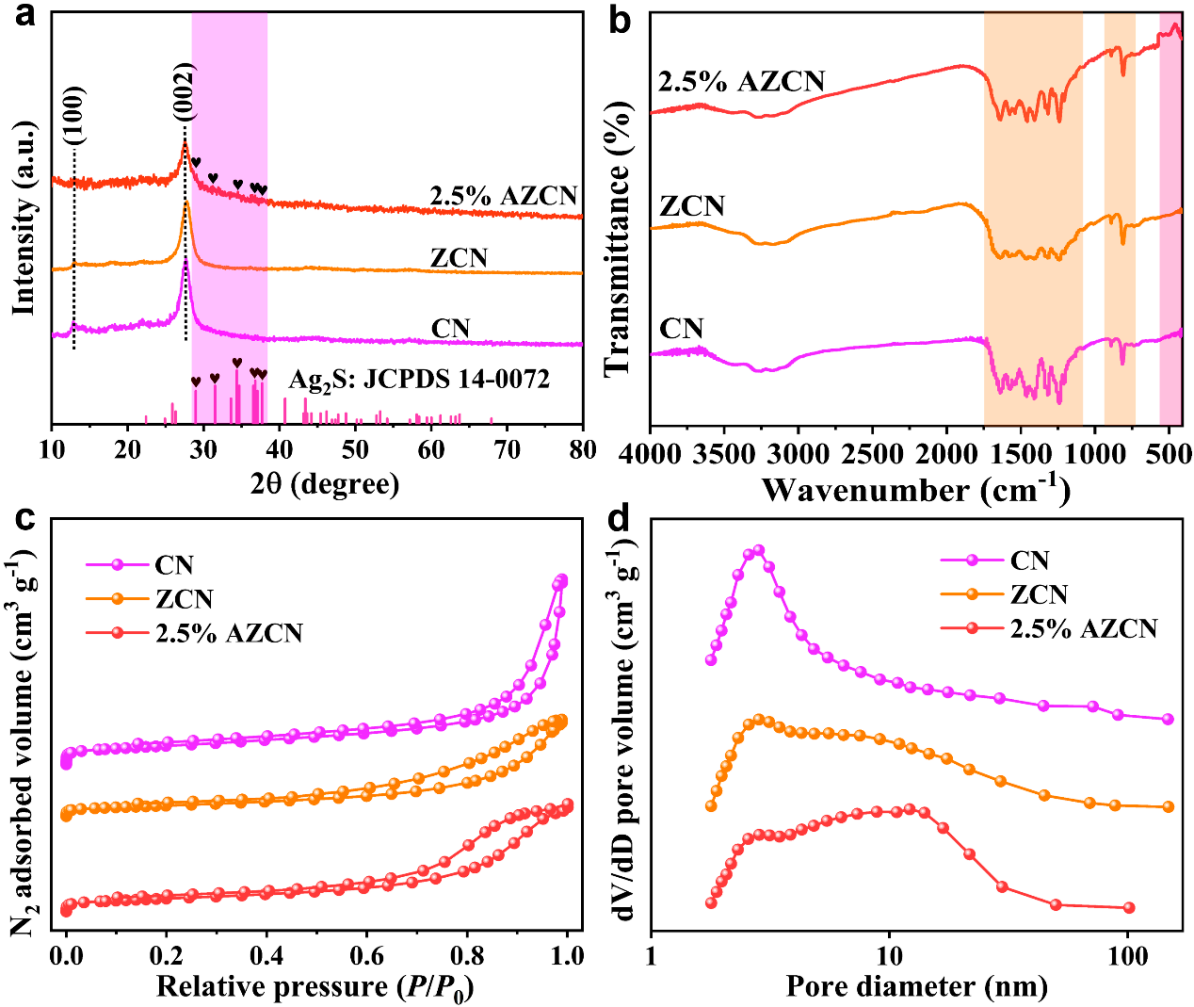
XRD curves (**a**), FT-IR spectra (**b**), N_2_ adsorption–desorption isotherms (**c**) as well as pore diameter distribution plots (**d**) of CN (purple), ZCN (orange) and 2.5% AZCN (red).

The crystalline phase of 2.5% AZCN was investigated using the XRD technique and compared with that of CN and ZCN. As presented in Figure 2a, similar to CN and ZCN, the optimal heterojunction delivers the characteristic peaks of g-C_3_N_4_, separately corresponding to the (002) and (100) planes [38]. Meanwhile, a series of weak peaks appear in the pattern of 2.5% AZCN, especially in the 2θ range of 28.5–38.5°, which are relevant to monoclinic phase Ag_2_S (JCPDS No. 14-0072) [39]. In addition, the (002) plane diffraction intensity decreases compared with CN and ZCN because of the introduction of Ag_2_S. The FT-IR analyses for these samples present a similar internal functional group structure. As provided in Figure 2b, apart from the broad IR absorption peak at about 3300 cm^−1^ attributed to the stretching vibration of the O-H bond in water physically absorbed on the sample surface, distinct absorption peaks in the regions of 1084–1737 cm^−1^ and 724–936 cm^−1^ are observed in their IR spectra. The former corresponds to the stretching vibrations of the C-N heterocycles, and the latter is relevant to tri-s-triazine (namely the respiratory vibration of the triazine unit) [40,41]. Furthermore, for 2.5% AZCN, the additional IR absorption peak relevant to the characteristic vibration of Ag-S is observed below 600 cm^−1^ [42], suggesting the successful introduction of Ag_2_S in ZCN.

The porous structure of 2.5% AZCN was investigated through a nitrogen adsorption/desorption experiment and compared with that of CN and ZCN. Compared with CN and ZCN, although having similar type-IV isotherms, the heterojunction displays a hysteresis loop with a larger integral area that is distributed in a lower relative pressure range (Figure 2c), suggesting more mesopores [43]. The pore diameter distribution plots further recommend the mesopore-dominant hierarchical porous structure of 2.5% AZCN (Figure 2d). As summarized in Table S1, although smaller than that of CN, the specific surface area (133.5 m^2^ g^−1^) and total pore volume (0.420 cm^3^ g^−1^) of 2.5% AZCN are obviously larger than that of ZCN (S_BET_ = 112.2 m^2^ g^−1^; V_p_ = 0.376 cm^3^ g^−1^). The main reason is that some Ag_2_S NPs accumulate in the submicron pores of ZCN, which effectively regulates the porosity, resulting in an increased detectable surface area and pore volume.

**Figure 3:**
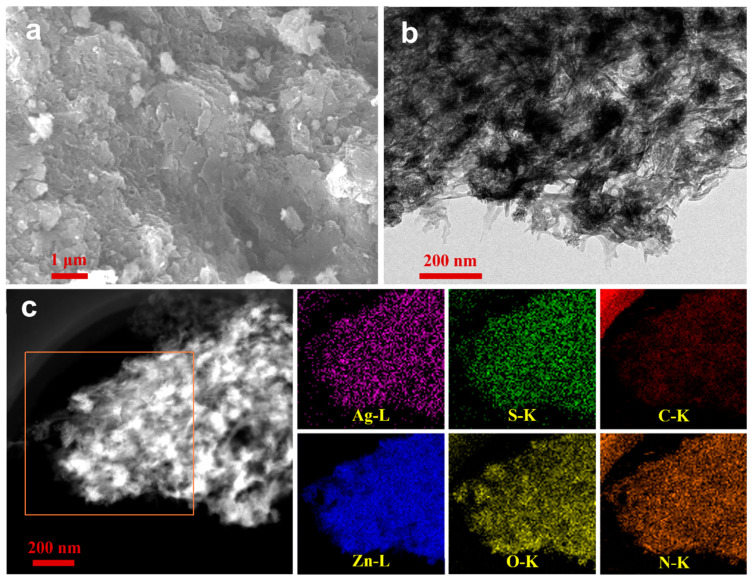
SEM image (**a**), TEM image (**b**) and EDX element mapping images (**c**) of 2.5% AZCN in the selected area (orange box).

The SEM image shown in Figure 3a shows that 2.5% AZCN poses a nanoplate structure with a rough surface, suggesting that this heterojunction maintains a relatively large surface area. Meanwhile, the TEM image shown in Figure 3b demonstrates that the surface structure of 2.5% AZCN has distinct areas of bright and dark, which signifies the 3D interconnected porous network of the pristine CN [24]. This unique porous structure with a large specific surface area favors incident light harvesting and increasing the adsorption capacity for reactant molecules, as well as the migration of photoinduced carriers to the material surface and mass transfer during photocatalytic reaction. In addition, the SEM image of Ag_2_S is shown in Figure S2, and the dispersion state of all elements is also identified via the EDX analysis. The EDX spectrum shown in Figure S3 and the element mapping images (Figure 3c) show that the relevant elements (namely Ag, S, Zn, C, N and O) exist evenly in the selected detection area. This verifies that the Ag_2_S NPs are evenly dispersed on ZCN sheets. Furthermore, apart from CN, the high-resolution TEM (HRTEM) image depicted in Figure S4 manifests the presences of ZnO (*d*_(100)_ = 0.282 nm) [44] and Ag_2_S (*d*_(121)_ = 0.248 nm) [40,45], identifying the construction of the proposed heterojunction material.

**Figure 4:**
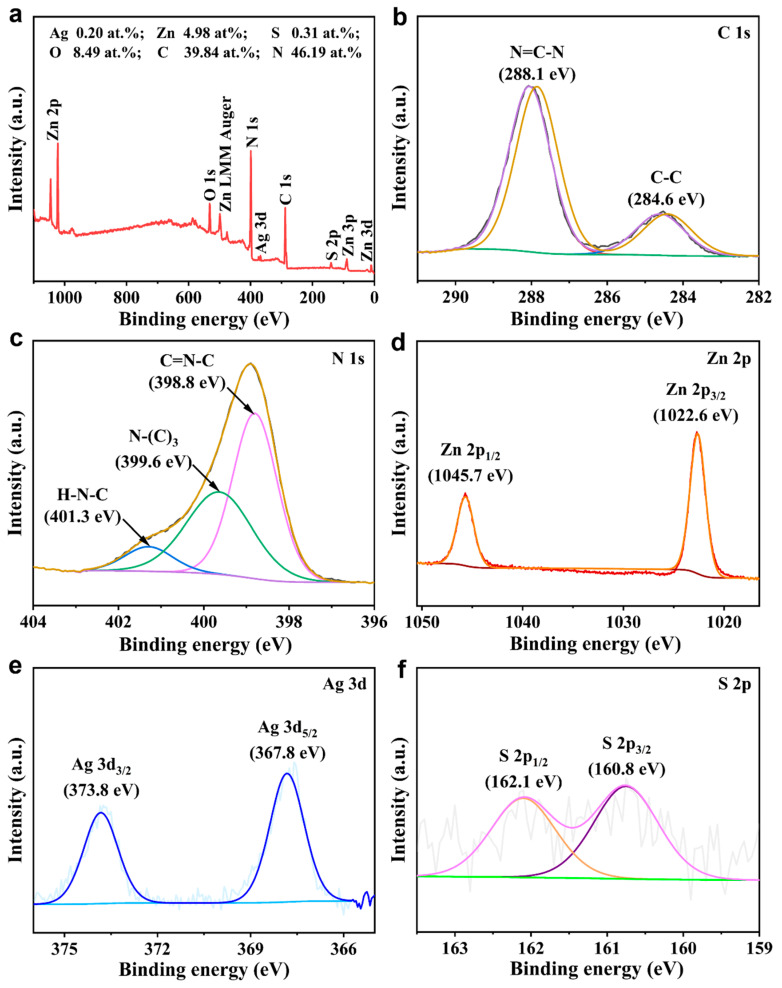
XPS full spectrum of 2.5% AZCN (**a**), as well as the relevant fine spectra of C 1s (**b**), N 1s (**c**), Zn 2p (**d**), Ag 3d (**e**) and S 2p (**f**).

Also, the XPS full spectrum shown in Figure 4a further indicates that 2.5% AZCN comprises the elements Ag, S, Zn, O, C and N. Specially, as shown in Figure 4b,c, apart from C-C, the C 1s and N 1s XPS fine spectra demonstrate that the C and N elements are involved in the bonds C-N=C, N-(C)_3_ and H-N-C, respectively [39]. The peaks at 1022.6 eV and 1045.7 eV in the Zn 2p XPS fine spectrum of 2.5% AZCN (Figure 4d) are separately attributed to Zn 2p_3/2_ and Zn 2p_1/2,_ suggesting that the metal element is still in the Zn^2+^ state in this heterojunction [46]. As expected, the corresponding fine spectra can directly prove the presence of Ag_2_S in 2.5% AZCN. Two individual peaks appear at 367.8 eV and 373.8 eV in the Ag 3d XPS fine spectra (Figure 4e), which are separately relevant to Ag 3d_5/2_ and Ag 3d_3/2_ [47]. The S 2p_3/2_ and S 2p_1/2_ signals are observed at 160.8 eV and 162.1 eV, respectively, in the S 2p XPS fine spectra (Figure 4f).

**Figure 5:**
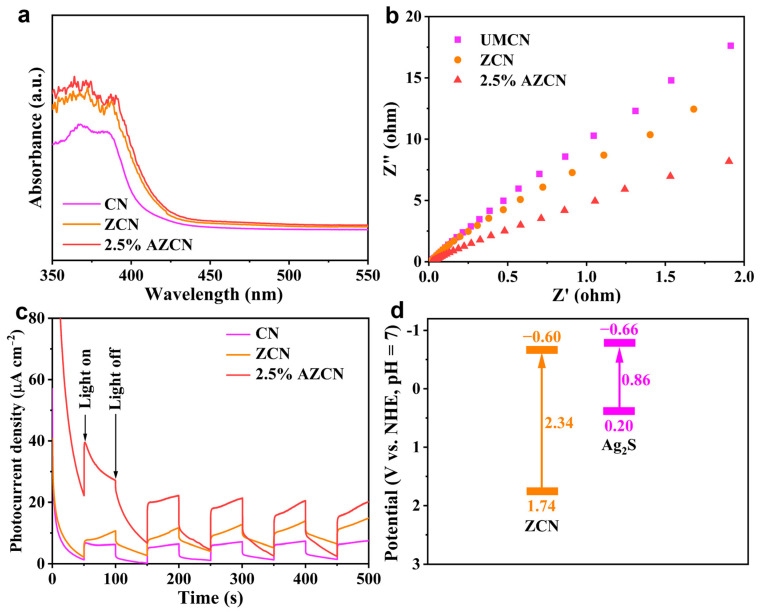
UV–Vis DRS curves (**a**), EIS curves (**b**) and transient photocurrent response curves (**c**) of CN, ZCN and 2.5% AZCN. The band structure diagram of ZCN and Ag_2_S (**d**).

The optical properties of 2.5% AZCN were explored using the ultraviolet–visible (UV–Vis) diffuse reflection spectroscopy (DRS) method and compared with that of CN and ZCN. As depicted in Figure 5a, the light absorption capacity of the as-prepared heterojunction is higher than that of CN and ZCN. Meanwhile, the absorption band edge of 2.5% AZCN is further redshifted slightly compared to ZCN because of the introduction of Ag_2_S posing a strong spectrum response even in the near IR region (Figure S5a). The electrochemical impedance spectroscopy (EIS) curves presented in Figure 5b reveal that 2.5% AZCN depicts a smaller arc radius than CN and ZCN, suggesting a lower charge-transfer resistance. Their transient photocurrent response plots verify that 2.5% AZCN displays the strongest photocurrent response upon illumination among the three investigated samples (Figure 5c), signifying the highest influx of photogenerated electrons into the circuit [48]. The results propose that the coupling of Ag_2_S and ZCN favors the separation of photoinduced electron–hole pairs and charge transport, thus increasing the photocatalytic activity. The (Ahν)^1/2^ vs. hν plot depicted in Figure S5b indicates that the band gap of Ag_2_S is 0.86 eV, according to the Tauc method [49], while the Mott–Schottky plot illustrated in Figure S5c reveals that the flat band potential of Ag_2_S is −0.76 V vs. Ag/AgCl, suggesting a conduction band energy (E_CB_) of −0.66 V vs. NHE. In addition, we have reported that the band gap and *E*_CB_ of ZCN are, separately, 2.34 eV and −0.60 V in our last work [25]. Hence, we drew the band structure diagram of ZCN and Ag_2_S (Figure 5d). Evidently, they have staggered energy band structures to construct the type-II heterojunction, which inhibits the fast recombination of photoinduced carriers and promotes the generation of more reactive groups, therefore improving the photocatalytic activity.

**Figure 6:**
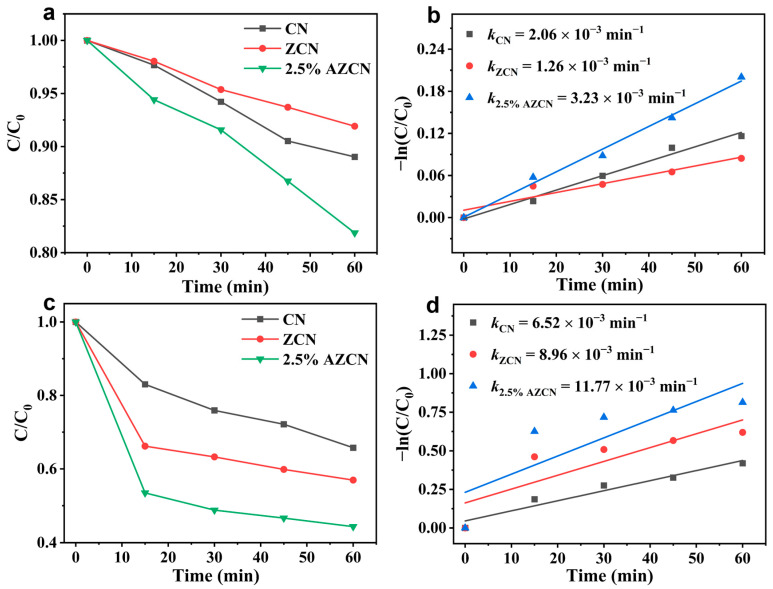
Photocatalytic activities of CN, ZCN and 2.5% AZCN (10 mg) toward the degradation of CIP (50 mL, 10 mg L^−1^) under NIR light illumination (using 700 nm cutoff filter) (**a**) and the corresponding curves of −ln(C/C_0_) vs. reaction time (**b**). Photocatalytic activities of CN, ZCN and 2.5% AZCN (10 mg) in the degradation of CIP (50 mL, 10 mg L^−1^) irradiated using simulated solar light (using an AM-1.5G filter) (**c**) and the corresponding curves of −ln(C/C_0_) vs. reaction time (**d**).

To verify the influence of different irradiation lights, apart from that of visible light, as discussed above (Figure 1), we further investigate the photocatalytic activity of 2.5% AZCN toward CIP degradation separately irradiated using NIR and simulated solar light and compared with that of CN and ZCN. Figure 6a presents that the degradation efficiency of CIP (18.1%) is 1.65 and 2.24 times that of CN (11.0%) and ZCN (8.1%) within 60 min under NIR light illumination, respectively. Also, as shown in Figure 6b, the *k* value over the optimal heterojunction (3.23 × 10^−3^ min^−1^) is higher than that over CN (2.06 × 10^−3^ min^−1^) and ZCN (1.26 × 10^−3^ min^−1^). When irradiated using the simulated solar light (Figure 6c,d), the photocatalytic activities of the three materials for CIP are remarkably improved compared to that of the NIR and visible lights, and 2.5% AZCN still provides the best performance under the same conditions. Catalyzed by the optimal heterojunction, the degradation efficiency of CIP and the corresponding *k* reach 55.7% within 60 min and 11.77 × 10^−3^ min^−1^. The results recommend that the optimal heterojunction is indeed broad-spectrum responsive toward CIP degradation.

**Figure 7:**
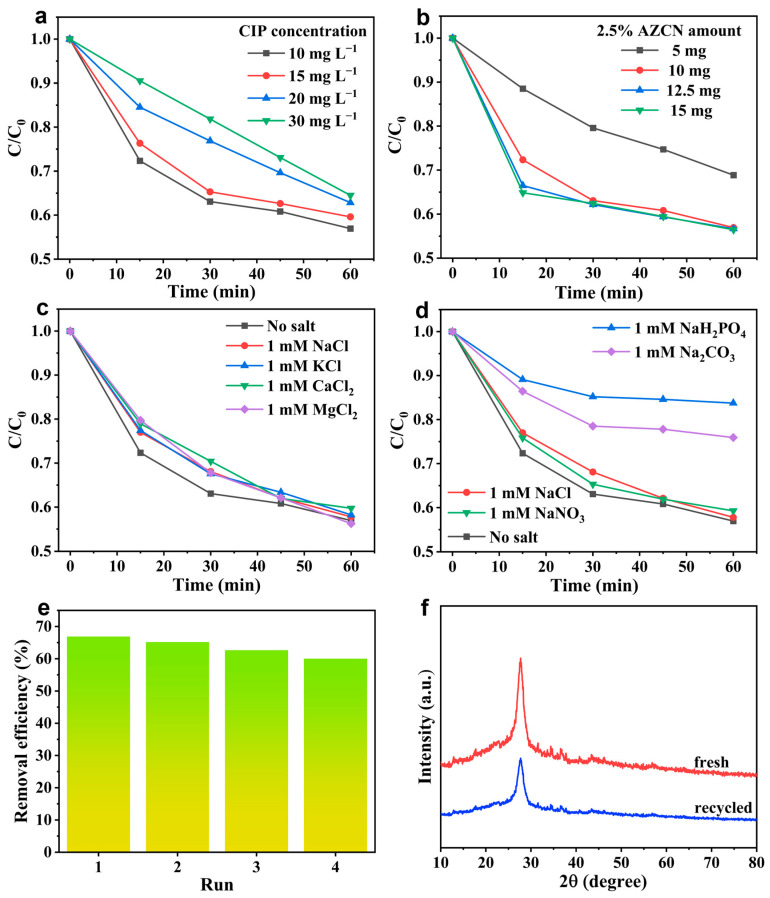
The influence of the initial pollutant concentration on the photocatalytic activity of 2.5% AZCN (10 mg) toward the degradation of CIP (50 mL) under visible light illumination (**a**). The effect of a 2.5% AZCN dosage on the photodegradation of CIP (50 mL, 10 mg L^−1^) under visible light illumination (**b**). The influence of different cations (**c**) and various anions (**d**) on the degradation of CIP (50 mL, 10 mg L^−1^) over 2.5% AZCN (10 mg) under visible light illumination. The cyclic stability of 2.5% AZCN (10 mg) in the photodegradation of CIP (50 mL, 10 mg L^−1^) separately irradiated by visible light for 120 min (**e**). XRD patterns of 2.5% AZCN before and after the cycling experiments (**f**).

Next, we further varied the photocatalytic reaction conditions to evaluate the universality of 2.5% AZCN in purifying CIP-based wastewater under visible light illumination. First, the CIP photodegradation experiment was performed without any catalyst under visible light, and the result indicates that the CIP concentration did not decrease, which proves that CIP cannot be degraded only via light irradiation (Figure S6). As demonstrated in Figure 7a, although CIP degradation decreases gradually with an increasing initial substrate concentration due to the limited amount of reactive sites on the surface of the specific amount of 2.5% AZCN and the limited number of free active radicals induced by the target system, the optimal heterojunction still delivers excellent photocatalytic activity even when the concentration of CIP is as high as 30 mg L^−1^, and the corresponding degradation is 35.5% within 60 min. Figure 7b presents that CIP degradation is significantly improved when increasing the amount of 2.5% AZCN from 5 mg to 10 mg, owing to the presence of more surface active sites and free radicals for the photocatalytic reaction. However, the degradation efficiency of increased CIP is no longer obvious when the photocatalyst amount is further increased to 12.5 mg and even decreases when the 2.5% AZCN dosage is increased to 15 mg. This is because the over-added 2.5% AZCN increases the turbidity and opacity of the CIP solution, partially hindering the refraction of the incident light [50]. Therefore, based on the purpose of controlling a proper reaction rate and the savings concept, we used 10 mg of 2.5% AZCN to catalyze the degradation of CIP (50 mL, 10 mg L^−1^) in the subsequent study.

Considering the possible co-existence of soluble inorganic species in real wastewaters, we separately introduced different chlorides (i.e., NaCl, KCl, CaCl_2_ and MgCl_2_) and sodium salts (i.e., NaCl, NaNO_3_, NaH_2_PO_4_ and Na_2_CO_3_) in the 2.5% AZCN-catalyzed CIP degradation system under visible light illumination. As presented in Figure 7c,d, compared to the control experiment, the effects of all chlorides and NaNO_3_ on the CIP degradation efficiency are ignorable, revealing that the obtained heterojunction photocatalyst is applicable in the degradation of CIP in water simultaneously containing these inorganic species. However, as shown in Figure 7d, the degradation efficiency of CIP decreases when Na_2_CO_3_ and NaH_2_PO_4_ are introduced. The inhibited degradation is primarily because H_2_PO_4_^−^ and HCO_3_^−^ induced from CO_3_^2−^ by reacting with H^+^ in the solution consume the reactive species (e.g., the photoproduced h^+^ and ⋅OH) [51,52].

To study the cyclic stability of 2.5% AZCN, we performed the cycling experiments for CIP degradation under visible light illumination, and the results are presented in Figure 7e. This heterojunction preserves relatively high photocatalytic activity even in the fourth run, with a degradation efficiency of 60.0%, suggesting favorable cyclic stability. Meanwhile, as shown in Figure 7f, the recycled 2.5% AZCN presents similar XRD diffraction characteristics to that of the fresh sample, evidencing desirable structural stability.

**Figure 8:**
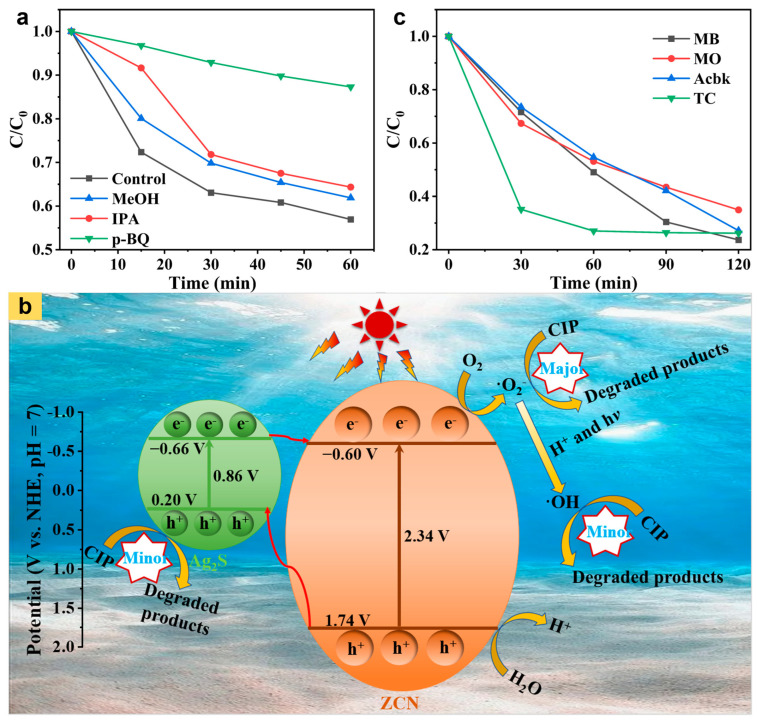
The free radical scavenging experiment results for the degradation of CIP (50 mL, 10 mg L^−1^) catalyzed by 2.5% AZCN (10 mg) under visible light illumination (MeOH/IPA 1 mL or p-BQ 1 mg) (**a**). The plausible photocatalytic mechanism of 2.5% AZCN for CIP degradation (**b**). Photocatalytic activity of 2.5% AZCN (10 mg) toward the degradation of other organic contaminants in water (50 mL, 10 mg L^−1^) under visible light illumination (**c**).

To identify the active free radicals in CIP degradation catalyzed by 2.5% AZCN under visible light illumination, we employed the scavenging experiments by introducing p-BQ, IPA and MeOH in the photocatalytic reaction system, respectively. As depicted in Figure 8a, compared to the control case, the introduction of p-BQ strikingly diminishes the degradation efficiency of CIP, suggesting that ⋅O_2_^−^ is the dominant active free radical in CIP degradation [53]. Meanwhile, the degradation of CIP is restricted to some extent when introducing MeOH and IPA, suggesting that ⋅OH and h^+^ also contribute to CIP degradation under the present conditions [54], but their effect is relatively small.

Based on the above scavenging experiment results, as well as the band structure diagram (Figure 5d), we propose the plausible photocatalytic mechanism of 2.5% AZCN for CIP degradation (Figure 8b and Equations (1)–(10)). Firstly, both Ag_2_S and ZCN absorb photons, and the photoinduced electrons shift from the corresponding valence band (VB) to the conduction band (CB), leaving photoinduced holes on VB, respectively (Equations (1) and (2)). The electrons on the CB of Ag_2_S tend to transfer to the CB of ZCN because of the redox potential difference. In turn, part of the holes on VB of ZCN migrate to the VB of Ag_2_S. Therefore, the migration facilitates the fast separation of the photogenerated carriers. The electrons on CB of ZCN are trapped by the O_2_ molecules solvated in the CIP solution to O_2_^−^ due to the more negative E_CB_ of ZCN (−0.60 V) than the O_2_/⋅O_2_^−^ reduction potential (−0.33 V vs. NHE) [55] (Equation (3)), and the residual holes on VB of ZCN react with the H_2_O molecules to produce H^+^ ions (Equation (4)). The ⋅OH free radicals are induced from H^+^ and partial ⋅O_2_^−^ under proper light illumination through Equations (5)–(7). The CIP molecules adsorbed on the heterojunction surface are attacked by ⋅O_2_^−^, causing the gradual degradation (Equation (8)), and this process poses a major role. Likewise, the produced ⋅OH and the holes on VB of Ag_2_S attack the adsorbed CIP molecules, further contributing to the substrate degradation (Equations (9) and (10)). Obviously, the construction of a type-II heterojunction with a suitable potential difference and unique porous structure contributes to the satisfactory photocatalytic performance of 2.5% AZCN in CIP degradation.

Finally, we further applied 2.5% AZCN in the photodegradation of other organic contaminants (i.e., MB, MO, Acbk and TC) in water under visible light illumination, and the results are illustrated in Figure 8c. Encouragingly, this heterojunction demonstrates remarkable photocatalytic activity toward the degradation of these pollutants. For example, the degradation efficiency of TC can reach 64.9% within 30 min, and the values of the MB, Acbk and MO dyes are 76.3%, 72.9% and 65.1% within 120 min, respectively. That is, 2.5% AZCN is effective in purifying wastewaters containing organic dyes and antibiotics, further attesting its excellent universality.

ZCN + hν ⟶ e^−^_CB_ (ZCN) + h^+^_VB_ (ZCN) (1)

Ag_2_S + hν ⟶ e^−^_CB_ (Ag_2_S) + h^+^_VB_ (Ag_2_S) (2)

O_2_ + e^−^_CB_ (ZCN) ⟶ ⋅O_2_^−^ (3)

2H_2_O + 4 h^+^_VB_ (ZCN) ⟶ 4H^+^ + O_2_ (4)

⋅O_2_^−^ + H^+^ ⟶ ⋅HO_2_ (5)

2⋅HO_2_ ⟶ O_2_ + H_2_O_2_ (6)

H_2_O_2_ + hν ⟶ 2⋅OH (7)

CIP + ⋅O_2_^−^ ⟶ Degraded products (CO_2_ + H_2_O) (8)

CIP + h^+^_VB_ (Ag_2_S) ⟶ Degraded products (CO_2_ + H_2_O) (9)

CIP + ⋅OH ⟶ Degraded products (CO_2_ + H_2_O) (10)

## 3. Experimental Methods

### 3.1. Photocatalyst Synthesis

**Scheme 1:**
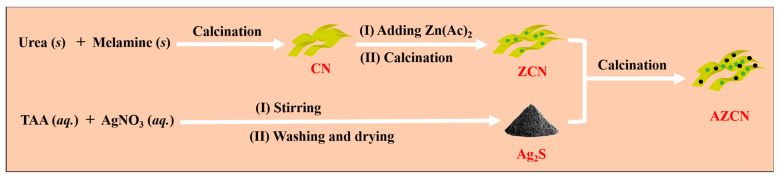
Schematic depiction of the preparation process for AZCN.

*Preparation of ZCN nanosheets*: Firstly, CN was prepared from urea and melamine using the co-polymerization method (550 °C, 4 h) [24]. Then, ZCN was synthesized via the sonication–calcination procedure [25] (Scheme 1). Briefly, Zn(Ac)_2_ (0.0297 g) was dissolved in ultrapure water (60 mL) and mixed with CN (0.1 g). After sonicating for 1 h and stirring for 24 h at room temperature, the solvents were gradually evaporated at 75 °C under stirring. Subsequently, ZCN was obtained by calcinating the residuals at 400 °C for 4 h in a muffle furnace.

*Preparation of Ag_2_S NPs*: Ag_2_S NPs were prepared via a conventional chemical precipitation method (Scheme 1). TAA (0.0375 g) and AgNO_3_ (0.0850 g) were separately dissolved in ultrapure water (50 mL) under stirring. Afterward, the resulting AgNO_3_ solution was dropped into the TAA solution and kept stirring for 2 h. The black Ag_2_S particles were collected via centrifugation after separately washing with ultrapure water and anhydrous ethanol six times and dried at 60 °C.

*Preparation of AZCN heterojunctions*: The heterojunctions were prepared by directly calcinating the mixture of ZCN and Ag_2_S (Scheme 1). First, specific amounts of ZCN and Ag_2_S were mixed and ground evenly. Then, the mixture was calcinated at 400 °C for 4 h. The as-prepared materials were labeled as *x*% AZCN (*x* is the mass percentage of Ag_2_S in the as-prepared heterojunctions; *x* = 1.0, 2.5 and 5.0).

### 3.2. Photocatalytic Experiments

*The optimal heterojunction screening experiments*: The activity difference among CN, ZCN and *x*% AZCN (10 mg) was evaluated in the degradation of CIP (50 mL, 10 mg L^−1^) irradiated using visible light. To reach the adsorption–desorption equilibrium, the feeding photocatalyst and CIP solution were firstly stirred for 60 min in the dark. Subsequently, a PLS-SXE300+/300 + UV xenon lamp (Perfectlight Technology Co., Ltd, Beijing, China) was turned on to trigger the photocatalytic reaction under visible light illumination. Then, 5 mL of the suspension was drawn out at 15 min intervals and centrifugally treated. Finally, the CIP concentration in the resulting solution was measured with a TU-1901 UV–Vis spectrophotometer (Purkinje GENERAL Instrument Co., Ltd, Beijing, China) (λ = 275 nm). The standard curves of CIP with different concentrations are shown in Figure S7.

*Cyclic experiments:* The cyclic stability of the optimal heterojunction (10 mg) was investigated by performing a series of degradation experiments for CIP (50 mL, 10 mg L^−1^) under visible light illumination. The reaction time was 120 min for every run. Before use in the subsequent experiment and for conducting XRD analysis, the feeding heterojunction was filtered, washed with ultrapure water and anhydrous ethanol and dried at 60 °C.

*The universality verification experiments*: The universality of the optimal heterojunction was studied by performing a range of CIP photocatalytic degradation experiments by adjusting certain reaction parameters (i.e., irradiated light type, contaminant concentration, optimal heterojunction dosage and co-existing anion or cation). To be noted, the simulated solar light, visible light and near infrared (NIR) light were directly offered by the xenon lamp or by equipping with a 420 nm and 700 nm cutoff filters, respectively. The detailed conditions can be found in the relevant figures or figure captions. Accordingly, the characteristic wavelengths in the UV–Vis absorption analyses for other contaminants were separately controlled as MB 664 nm, MO 464 nm, Acbk, 522 nm and TC 276 nm.

*Free radical scavenging experiments*: To verify the possible active species, a series of photodegradation experiments of CIP (50 mL, 10 mg L^−1^) over 2.5% AZCN (10 mg) were performed under visible light illumination by using the scavengers of methanol (MeOH, 1 mL), isopropanol (IPA, 1 mL) and p-benzoquinone (p-BQ, 1 mg) for the photoinduced hole (h^+^), hydroxyl radical (⋅OH) and superoxide radical (⋅O_2_^−^), respectively.

## 4. Conclusions

In summary, Ag_2_S nanoparticles are facilely coupled with Zn^2+^-decorated graphitic carbon nitride (ZCN) to prepare the type-II heterojunction AZCN. The 3D interconnected porous structure with a large specific surface area and the synergistic heterojunction effect of Ag_2_S and ZCN favor the utilization of incident light and mass transfer, enhance the adsorption capacity for reactant molecules and promote the separation and transport of the photoproduced carriers. Therefore, the resulting materials, especially the optimal 2.5% AZCN, present excellent photocatalytic performance and exciting universality in the degradation of various organic contaminants (such as CIP, TC, MO, MB and Acbk). This study develops an effective type-II heterojunction photocatalyst and proposes valuable insights for water remediation via the photocatalytic strategy.

## Figures and Tables

**Table 1:** CIP degradation efficiencies in the presence of different photocatalysts, published recently.

Photocatalyst	CIP Concentration (mg L^−1^)	Photocatalyst Dosage (g L^−1^)	Light Type	Irradiation Time (min)	CIP Elimination Efficiency (%)	Ref.
Gd@Bi_2_MoO_6_/rGO	5	0.40	simulated solar light	120	81.7	[6]
UCN-1 h	2.5	0.50	simulated solar light	90	98.8	[19]
CNNS/NH_4_V_4_O_10_	10	0.50	simulated solar light	100	92.0	[20]
V_2_O_5_/g-C_3_N_4_	10	1.00	visible light	180	91.0	[22]
F-BiVO_4_/g-C_3_N_4_/CdS	20	1.00	simulated solar light	30	90.0	[26]
KPF_6_-g-C_3_N_4_	10	0.40	visible light	180	90.8	[27]
LaFeO_3_/g-C_3_N_4_/Ag_3_PO_4_	10	0.75	visible light	120	90.2	[28]
Cs_3_PMo_12_O_40_/MnIn_2_S_4_	20	1.00	visible light	60	61.8	[29]
CdS QDs@MOF-808	40	0.20	visible light	180	82.0	[30]
Pt/BaTiO_3_/Bi_2_O_3_	10	1.00	simulated solar light	60	63.0	[31]
Fe_3_O_4_/ZnO/Lys-rGO	20	0.25	visible light	60	38.0	[32]
In_2_O_3_/BiOBr	10	0.50	visible light	90	93.5	[33]
BC_15_@FeTiO	10	0.20	visible light	120	91.1	[34]
MoS_2_/V_2_O_5_	20	0.40	visible light	120	98.7	[35]
BiOCOOH/Bi_2_MoO_6_	20	0.50	visible light	120	95.6	[36]
DyCrO_3_	12	0.80	simulated solar light	240	83.0	[37]
2.5% AZCN	10	0.20	visible light	60	43.1%	This study

## Data Availability

No new data were created or analyzed in this study.

## Supplementary Materials

The following supporting information can be downloaded at https://www.mdpi.com/article/10.3390/molecules30071417/s1: Figure S1: The adsorption of CIP experiment under dark condition; Figure S2: TEM image of Ag_2_S; Figure S3: EDX spectrum of 2.5% AZCN; Figure S4: HRTEM image of 2.5% AZCN; Figure S5: UV-vis-IR DRS curve, (Ahν)^1/2^ vs. hν plot and Mott-Schottky plot of Ag_2_S; Figure S6: The degradation of CIP under visible light; Figure S7: The absorbance spectra of CIP in 200 nm to 400 nm; Table S1: The values of S_BET_ and V_p_ of CN, ZCN and 2.5% AZCN.
