# Supplementary material for: Ag2S/Zn2+-Decorated g-C3N4 Type-II Heterojunction with Wide-Spectrum Response: Construction and Photocatalytic Performance in Ciprofloxacin Degradation
Source: Molecules. 2025 Mar 22;30(7):1417. doi: 10.3390/molecules30071417 (PMC11990859; doi:10.3390/molecules30071417)
Supplement: Supplementary file 1 [file molecules-30-01417-s001.zip › molecules-3506581-supplementary.pdf]

Supplementary Material

Ag_2S/Zn^{2+}-Decorated g-C_3N_4 Type-II Heterojunction with Wide-Spectrum Response: Construction and Photocatalytic Performance in Ciprofloxacin Degradation

Chengyang Wang [1,†], Han Zheng [1,†], Ruxue Ma [1], Xiucheng Zheng [1,2,*] and Xinxin Guan [1,*]

[1] College of Chemistry, Zhengzhou University, Zhengzhou 450001, China
[2] State Key Laboratory of Coking Coal Resources Green Exploitation, Zhengzhou University, Zhengzhou 450001, China
* Correspondence: zhxch@zzu.edu.cn (X.Z.); guanxin@zzu.edu.cn (X.G.)
† These authors contributed equally to this work.

Reagents

Silver nitrate ($AgNO_3$), melamine, isopropanol (IPA), p-benzoquinone (p-BQ) and methanol (MeOH) were produced by Sinopharm Chemical Reagent Co., Ltd. Zinc acetate ($Zn(Ac)_2$) was produced by Aladdin Reagent (Shanghai) Co., Ltd. Urea was produced by Tianjin Windship Chemical Reagent Technology Co., Ltd. Thioacetamide (TAA), tetracycline (TC) and methyl orange (MO) were produced by Tianjin Comio Chemical Reagent Co., Ltd. Ciprofloxacin (CIP) was produced by Guangzhou Baiyunshan Pharmaceutical General Factory. MB was produced by China Pharmaceutical Corporation Beijing Purchasing Supply Station. Acid chrome blue K (Acbk) was produced by East China Normal University Chemical Factory. All reagents were of analytical grade and used upon receipt. Ultrapure water with a resistivity of 18.25 MΩ cm was produced with the 1810v Molecular water treatment equipment.

Characterization

The crystalline phases of the samples were characterized on a Panalytical X' pertPro diffractometer (CuK_a radiation, λ = 1.54 Å). The working voltage and current of the X-ray tube were 40 kV and 40 mA, respectively.

The porous parameters of the samples were measured with a Micromeritics ASAP 2420-4MP accelerated surface area and porosimetry system. The specific surface area (S_{BET}) was measured with the Brunauer-Emmett-Teller (BET) method, and the total pore volume (V_p) was evaluated at the relative pressure of 0.99. The pore width distribution curve was acquired from the adsorption branches based on the Barret-Joyner-Halenda (BJH) method. Before the N_2 adsorption-desorption experiments, the

samples (about 0.57 g) were degassed at 150 °C for 3 h under vacuum.

The morphology and microstructure of the samples were observed with a Sigma 500 field emission scanning electron microscope (SEM) operated at 5 kV and a Tecnai G2 F20 S-Twin transmission electron microscope (TEM) at 200 kV.

The surface chemical information of the samples was investigated on a Shimadzu AXIS-UL TRA DLD X-ray photoelectron spectrometer (XPS). The C 1s photoelectron line (binding energy = 284.6 eV) was used to calibrate the binding energies of the photoelectron.

The UV-vis diffuse reflectance spectra (DRS) curves of CN, ZCN and 2.5% AZCN were recorded on a Persee TU-1901 spectrometer, and UV-vis-IR DRS curve of Ag_2S were recorded on a Cary 5000 Agilent UV-Vis-NIR spectrometer. Barium sulfate ($BaSO_4$) was used as the reference.

Fourier transform infrared (FT-IR) spectra of the samples were recorded on a Thermo Scientific Nicolet 380 FT-IR spectrometer by using the potassium bromide (KBr) pellet technique.

The photocurrent experiments and electrochemical impedance spectroscopy (EIS) experiments were performed on the CHI 760E electrochemical workstation with Na_2SO_4 solution (0.5 M) as the electrolyte under visible light irradiation (300 W Xe-lamp, wavelength range 420 - 780 nm). A platinum plate and an Ag/AgCl electrode acted as the counter and reference electrodes, respectively. The working electrode was prepared as follows: 10 mg of the samples were ultrasonically dispersed in a mixed solution of absolute ethanol (125 μL), ultrapure water (375 μL) and nafion (50 μL).

Then, the above suspension was dropped on a FTO glass ($1 \times 1\ cm^2$) and dried under infrared light irradiation.

Photocatalytic activity evaluation

The contaminant degradation efficiency and the corresponding reaction rate constant (k) are calculated according to ***Eqs.*** **S1** and **S2**, respectively. In the equations, C_0 and A_0 are the contaminant concentration and absorbance after reaching adsorption-desorption equilibrium, respectively; While C and A are separately the contaminant concentration and absorbance after a certain irradiation time.

$$\text{Degradation efficiency (\%)} = \frac{C_0 - C}{C_0} \times 100\% = \frac{A_0 - A}{A_0} \times 100\% \quad (S1)$$

$$\ln\left(\frac{C}{C_0}\right) = -kt \quad (S2)$$

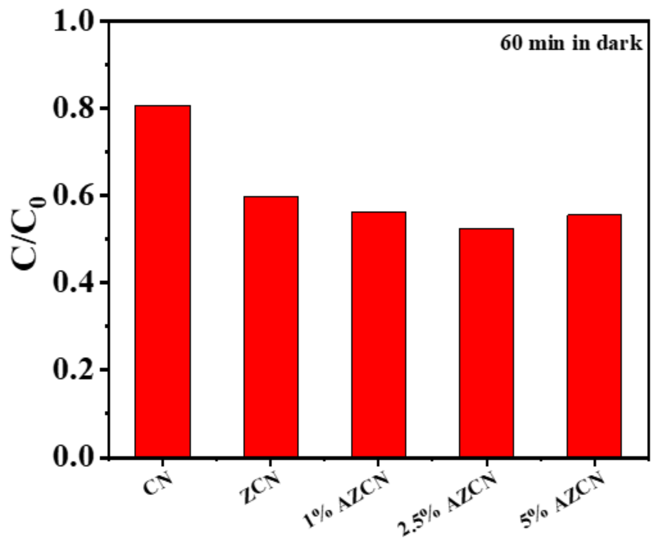

Figure S1. The adsorption of CIP experiment under dark condition.

Table S1. The values of S_{BET} and V_p of CN [S1], ZCN [S2] and 2.5% AZCN.

Sample	S_{BET} (m^2 g^{-1})	V_p (cm^3 g^{-1})
CN	183.7	0.724
ZCN	112.2	0.376
2.5% AZCN	133.5	0.420

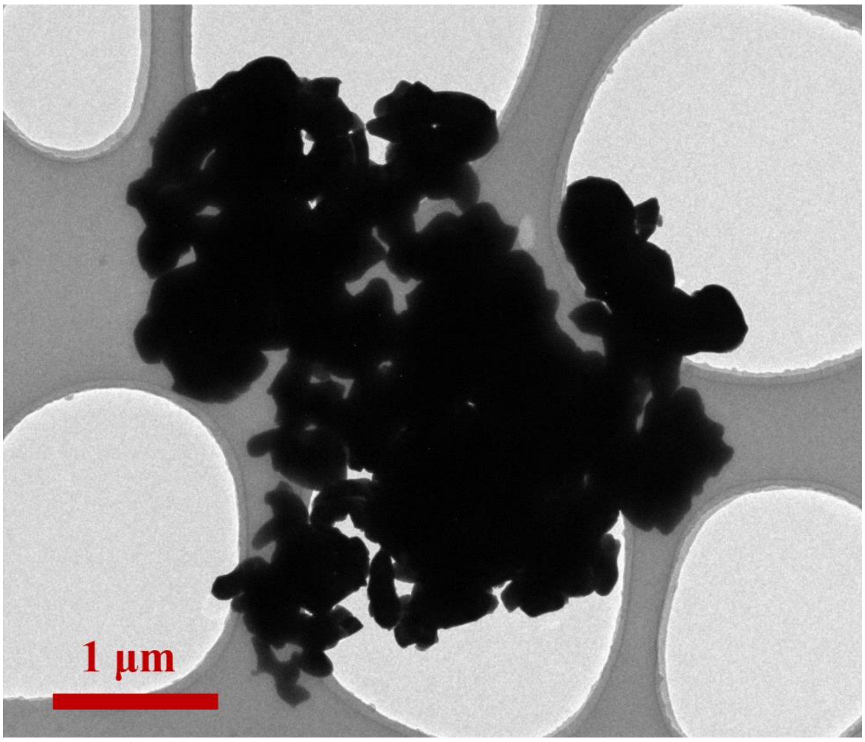

Figure S2. TEM image of Ag_2S.

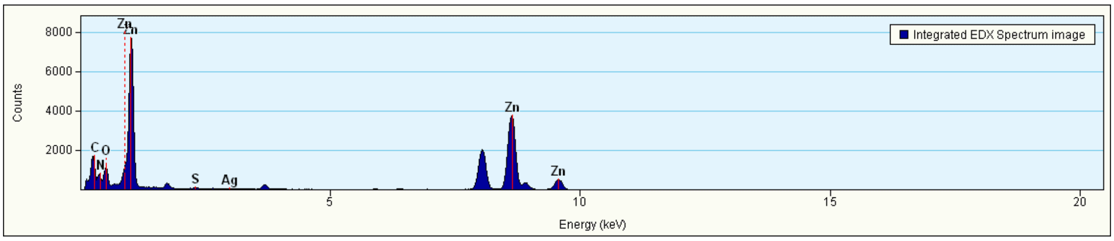

Figure S3. EDX spectrum of 2.5% AZCN.

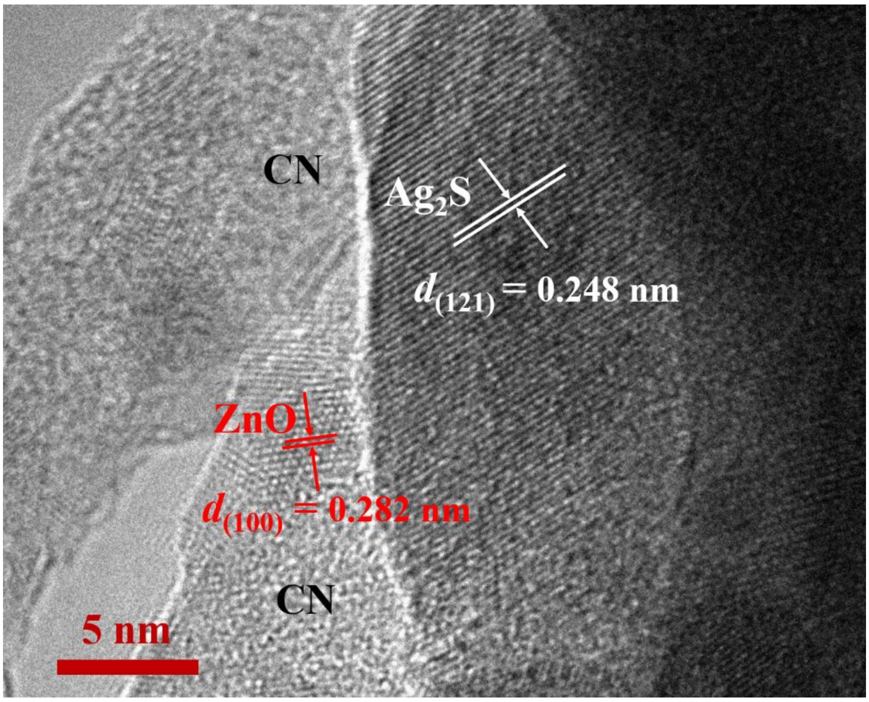

Figure S4. HRTEM image of 2.5% AZCN.

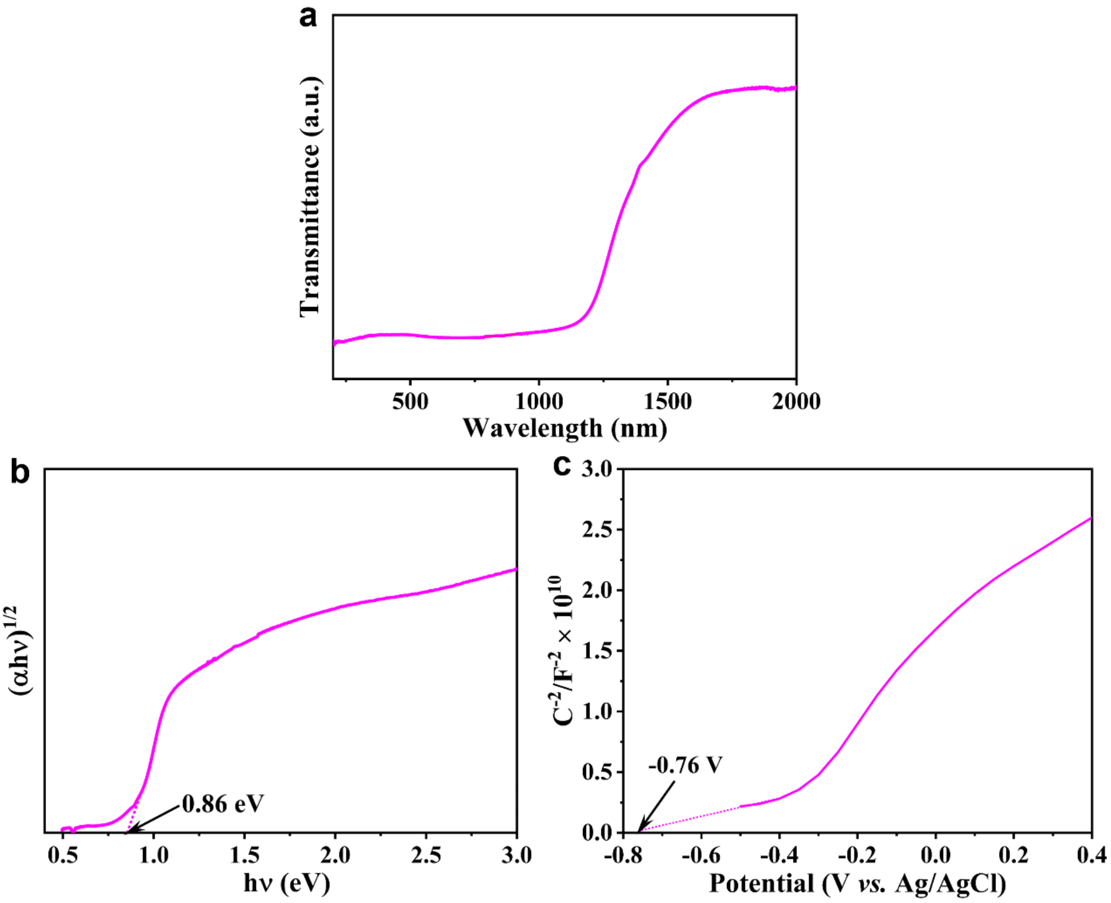

Figure S5. UV-vis-IR DRS curve (a), $(Ah\nu)^{1/2}$ *vs.* hν plot (b) and Mott-Schottky plot (c) of Ag_2S.

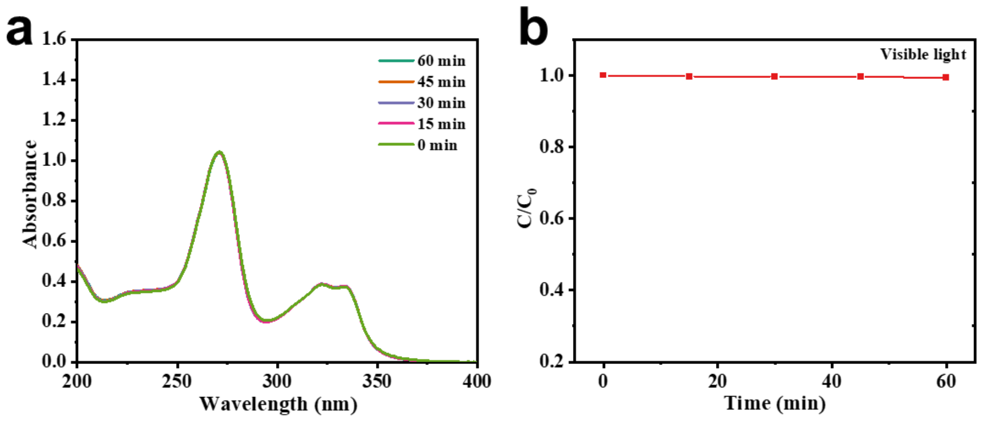

Figure S6. The self-degradation of CIP in the absence of catalyst under visible light (> 420 nm).

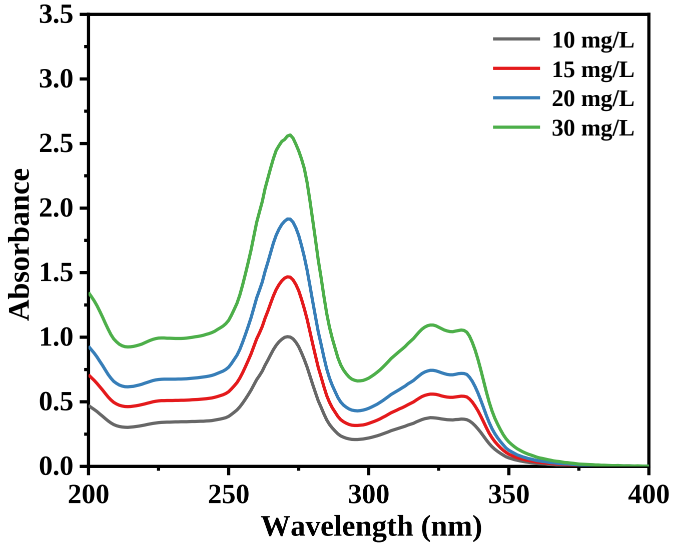

Figure S7. The absorbance spectra of CIP with different concentrations in the region of 200-400 nm.

References

[S1] H. Zheng, Y. Chen, X.F. Sun, X.C. Zheng, X.L. Zhang, X.X. Guan, Enhanced photocatalytic performance and mechanism of N-deficiently porous g-C_3N_4 in organic pollutant degradation, Mater. Res. Bull. 169 (2024) 112510. https://doi.org/10.1016/j.materresbull.2023.112510.

[S2] R.X. Ma, H. Zheng, J. Wang, X.C. Zheng, X.L. Zhang, X.X. Guan, Zn^{2+}-decorated

porous g-C_3N_4 with nitrogen vacancies: Synthesis, enhanced photocatalytic performance and mechanism in degrading organic contaminants, Mater. Res. Bull. 183 (2025) 113193. https://doi.org/10.1016/j.materresbull.2024.113193.
